# Maintenance Therapy for ATM-Deficient Pancreatic Cancer by Multiple DNA Damage Response Interferences after Platinum-Based Chemotherapy

**DOI:** 10.3390/cells9092110

**Published:** 2020-09-16

**Authors:** Elodie Roger, Johann Gout, Frank Arnold, Alica K. Beutel, Martin Müller, Alireza Abaei, Thomas F. E. Barth, Volker Rasche, Thomas Seufferlein, Lukas Perkhofer, Alexander Kleger

**Affiliations:** 1 Department of Internal Medicine 1, University Medical Center Ulm, Albert-Einstein-Allee 23, 89081 Ulm, Germany; elodie.roger@uni-ulm.de (E.R.); johann.gout@uni-ulm.de (J.G.); frank.arnold@uni-ulm.de (F.A.); alica.beutel@uni-ulm.de (A.K.B.); thomas.seufferlein@uni-ulm.de (T.S.); 2 Center for Translational Imaging (MoMAN), Ulm University, 89081 Ulm, Germany; alireza.abaei@uni-ulm.de (A.A.); Volker.rasche@uni-ulm.de (V.R.); 3 Institute of Pathology, University Medical Center Ulm, 89081 Ulm, Germany; thomas.barth@uniklinik-ulm.de

**Keywords:** pancreatic ductal adenocarcinoma, ATM, chromosomal instability, targeted therapy, DNA damage repair, platinum, PARP, maintenance therapy

## Abstract

Personalized medicine in treating pancreatic ductal adenocarcinoma (PDAC) is still in its infancy, albeit PDAC-related deaths are projected to rise over the next decade. Only recently, maintenance therapy with the PARP inhibitor olaparib showed improved progression-free survival in germline *BRCA1/2*-mutated PDAC patients after platinum-based induction for the first time. Transferability of such a concept to other DNA damage response (DDR) genes remains unclear. Here, we conducted a placebo-controlled, three-armed preclinical trial to evaluate the efficacy of multi-DDR interference (mDDRi) as maintenance therapy vs. continuous FOLFIRINOX treatment, implemented with orthotopically transplanted ATM-deficient PDAC cell lines. Kaplan–Meier analysis, cross-sectional imaging, histology, and in vitro analysis served as analytical readouts. Median overall survival was significantly longer in the mDDRi maintenance arm compared to the maintained FOLFIRINOX treatment. This survival benefit was mirrored in the highest DNA-damage load, accompanied by superior disease control and reduced metastatic burden. In vitro analysis suggests FOLFIRINOX-driven selection of invasive subclones, erased by subsequent mDDRi treatment. Collectively, this preclinical trial substantiates mDDRi in a maintenance setting as a novel therapeutic option and extends the concept to non-germline *BRCA1/2*-mutant PDAC.

## 1. Introduction

An early metastatic potential, aggressive and wasting tumor growth, and high therapy resistance are considered as hallmarks of pancreatic ductal adenocarcinoma (PDAC) [1]. In line, survival rates, especially at advanced tumor stages, have hardly improved over the last decades despite numerous clinical trials [2,3]. Consequently, median overall survival (mOS) in advanced disease rarely exceeds one year, with a global 5-year OS below 10%. Nowadays, the standard of care in treating advanced PDAC patients still refer to combinational approaches of various chemotherapeutic agents, culminating in FOLFIRINOX (folinic acid, fluorouracil, irinotecan, and oxaliplatin) as the most potent regimen [4,5]. Though, these therapeutic concepts do not take the molecular heterogeneity defining the PDAC subtypes into account, yet a paradigm shift towards targeted therapies as in other cancers has not occurred [3]. Indeed, albeit significant advances in molecular characterization of the different existing subtypes, the full spectrum of PDAC has yet to be fully captured [6]. New therapeutic concepts are urgently needed to effectively eradicate specific tumor subclones and, thus, improve patient outcomes. A particularly aggressive PDAC subtype, defined as unstable and frequently harboring mutations in DNA damage response (DDR) genes, such as *BRCA1/2* and *ATM*, is supposed to be sensitive toward DNA-damaging agents, such as platins [7]. Such deleterious DDR mutations occur as somatic but as germline mutations as well [8], with still elusive clinical consequences on patient treatment. Hence, the selective interference with the DDR is a promising approach as exemplified by the multiple ongoing clinical trials using DDR inhibitors [8,9], although mostly studying non-pancreatic cancers. Recently, the PARP inhibitor olaparib was the first targeted approach being effective in germline *BRCA1/2* (*gBRCA*) mutant PDAC, a mutation enriched in the so-called genomic unstable PDAC subtype [7]. Nevertheless, the underlying Phase III trial (POLO trial) revealed several obstacles (i) as it focused only on *gBRCA* mutations, (ii) implemented a rather unconventional maintenance arm with olaparib monotherapy vs. placebo after FOLFIRINOX induction, and (iii) resulted only in an improved progression-free survival (PFS) without improving median overall survival (mOS) [10]. Accordingly, first-line platinum-based chemotherapy has been applied to patients harboring both somatic and germline mutations in homology repair (HR) genes, leading to a favorable outcome in support of the POLO trial, thereby extending the concept to generally HR-deficient tumors, at least in terms of the PFS [11]. However, with an overall mutational frequency of approx. 4%, the *ATM serine/threonine kinase* (*ATM*) is the most frequently mutated DDR gene in sporadic PDAC, before the *BRCA1* and *BRCA2* genes [12]. *ATM* acts as a crucial enzyme in HR, its deficiency representing a bona fide HR-defective PDAC model for preclinical studies [13]. We could previously show that impaired *ATM* expression associates with a poor prognosis in human PDAC. *ATM* and *BRCA1/2* are key players in the DDR, exerting crucial steps during DNA double-strand break (DSB) repair by homologous recombination. Accordingly, the deletion of *Atm* in a mouse model of pancreatic cancer (*Atm^fl/fl^; LSL-Kras^G12D/+^; Ptf1a^Cre/+^*, termed AKC) also causes genomically unstable PDACs, featuring chromosomal instability, disruption of DNA integrity, and aneuploidy, which are indeed sensitive to single PARP inhibitor treatment [10,14,15,16]. However, preclinical studies showed only (i) moderate effectivity of olaparib as a single agent and (ii) indicated early chemoresistance in s.c. transplants of cell lines derived from tumor-bearing AKC mice [16]. Therefore, alternative therapy approaches are warranted to refine effectivity since, for instance, the combination of PARP inhibitors with chemotherapy is likely increasing toxicity [17] and not necessarily more efficient [18]. Thus, concepts that target compensatory, mainly HR-related pathways against PARP inhibition in an ATM-deficient PDAC appear reasonable [19]. Indeed, others and we found ATR (HR repair) and DNA-PKcs (non-homologous end joining, NHEJ) as corroborating pathways to ATM-signaling in PDAC [13,20,21]. In a recently published study, we showed that PARP, ATR, and DNA-PK inhibitors (PAD) act synergistically on ATM-deficient cells, leading to a lethal accumulation of DNA damage and, thus, demonstrating PAD treatment is a valuable approach that can be exploited to target *ATM*-mutant human PDACs effectively [13]. Consequently, we tested a putative synthetically lethal therapeutic option by multi-DDR interference (mDDRi) with PAD in a maintenance therapy setting after platinum-based induction therapy in analogy to the POLO trial for efficacy in ATM-deficient PDAC.

## 2. Materials and Methods

### 2.1. Mice and Ethics Statement

*Atm^fl/fl^*, *LSL-Kras^G12D/+^*, and *Ptf1a^Cre/+^* were previously described [22,23,24]. Eight-week-old female Hsd:Athymic Nude-*Foxn1^nu^* mice were purchased from Envigo (Indianapolis, IN, USA). Mice were housed and bred in a conventional health status-controlled animal facility. All animal care and procedures followed German legal regulations and were previously approved by the respective governmental review board of the state of Baden-Württemberg (permission no. 1369 and 1273). All the aspects of the mouse work were carried out following strict guidelines to insure careful, consistent, and ethical handling of mice.

### 2.2. Orthotopic Pancreatic Cancer Model

For orthotopic transplantations, cells were implanted by an injection of 50 × 10^3^ cells in 100 μL of 1:1 serum-free DMEM (ThermoFisher Scientific, Waltham, MA, USA): Matrigel GFR (Corning, NY, USA) into the pancreas of eight-week-old (24–26 g body weight) female Hsd:Athymic Nude-*Foxn1^nu^* mice (*n* = 8 for each condition). To circumvent any dominance issue, only Hsd:Athymic Nude-*Foxn1^nu^* females were used in this study.

**Figure 1:**
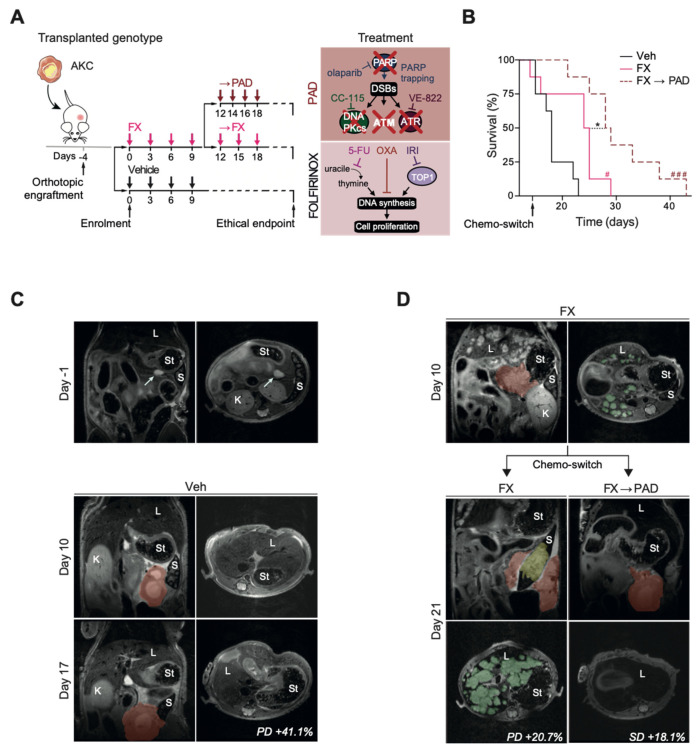
PAD maintenance therapy prolongs disease control in ATM-deficient PDAC after FOLFIRINOX induction. (**A**) Schematic representation of the orthotopic assay shown in (**B**) with respective treatment administration schedule. (**B**) Kaplan–Meier analysis of survival of the orthotopic assay performed on athymic Nude-*Foxn1^nu^* mice with *Atm^fl/fl^; LSL-Kras^G12D/+^; Ptf1a^Cre/+^* (AKC) cells, treated or not with FOLFIRINOX (FX, folinic acid 50.0 mg/kg, 5-fluorouracil 25.0 mg/kg, irinotecan 25.0 mg/kg, and oxaliplatin 2.5 mg/kg) and PAD (PARP inhibitor, ATR inhibitor and DNA-PK inhibitor; respectively, olaparib 50.0 mg/kg, VE-822 20.0 mg/kg, and CC-115 2.5 mg/kg). Magnetic resonance imaging of orthotopic PDAC tumor-bearing mice treated or not as in (**B**), at (**C**) Day -1 before treatment start, Day 10, and Day 17 after treatment start, and (**D**) Day 10 and Day 21 after treatment start. Red colored areas highlight the primary pancreatic tumors. Green areas highlight liver metastases. Yellow area highlights spleen invasion. DSB, double-strand break; K, kidney; L, liver; PD, progressive disease; S, spleen; SD, stable disease; St, stomach; Veh, vehicle. White arrows show AKC primary pancreatic tumor development. *, *p* < 0.05; #, *p* < 0.05 when compared to Veh arm; ###, *p* < 0.001 when compared to the Veh arm.

**Figure 2:**
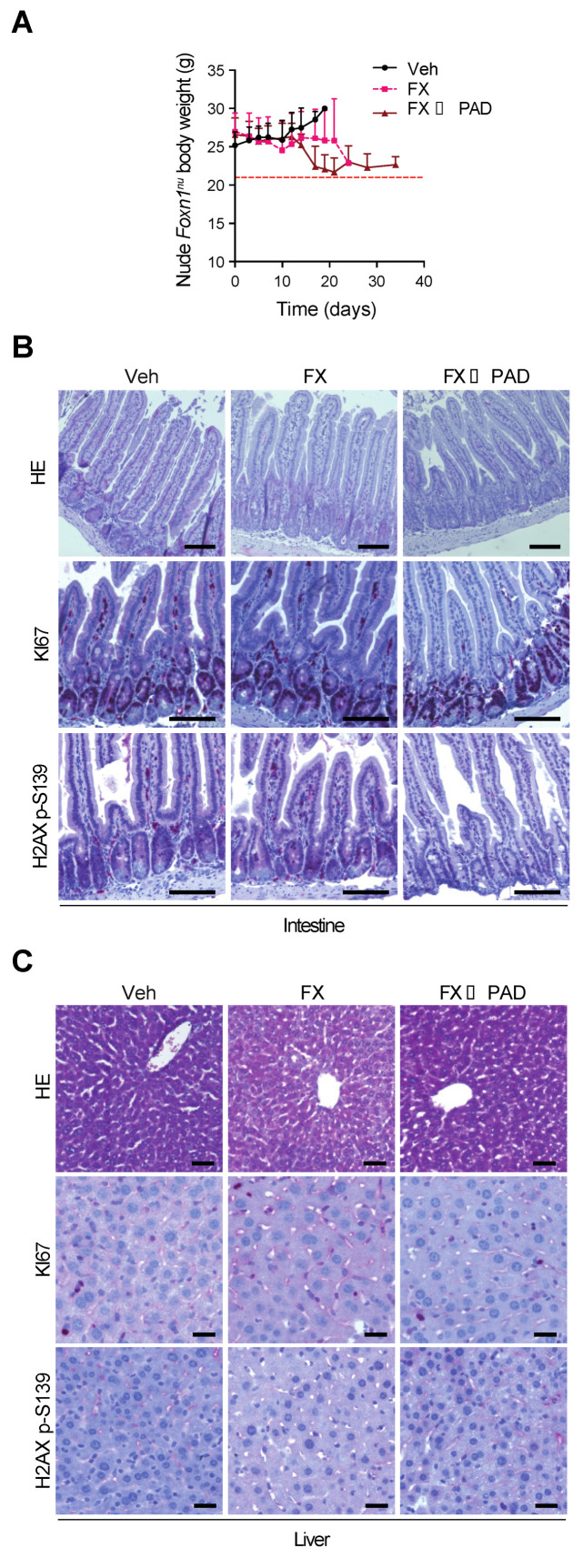
PAD maintenance and FOLFIRINOX therapy cause less adverse effects in athymic Nude-*Foxn1^nu^* mice. (**A**) Body weight progression of athymic Nude-*Foxn1^nu^* mice enrolled in the orthotopic assay shown in (Figure 1B). The horizontal red dashed line represents the body weight loss ethical endpoint (−20% of baseline weight). (**B**) Histologic sections stained by hematoxylin–eosin and immunohistochemistry staining for KI67 and H2AX p-S139 in resected intestines from the orthotopic assay shown in Figure 1B. Scale bars represent 100 µm. (**C**) Histologic sections stained by hematoxylin–eosin and immunohistochemistry staining for KI67 and H2AX p-S139 in resected livers from the orthotopic assay shown in Figure 1B. Scale bars represent 50 µm. FX, FOLFIRINOX; PAD, PARP inhibitor/ATR inhibitor/DNA-PK inhibitor; Veh, vehicle.

The solutions of olaparib, VE-822, and CC-115 (PAD: PARP inhibitor, ATR inhibitor, and DNA-PK inhibitor, respectively, 50.0 mg/kg, 20.0 mg/kg, and 2.5 mg/kg every second day), and FOLFIRINOX (folinic acid, 5-fluorouracil, irinotecan, and oxaliplatin, respectively, 50.0 mg/kg, 25.0 mg/kg, 25.0 mg/kg, and 2.5 mg/kg every third day) [25] were administered by i.p. injection. The treatment administration schedule is shown in Figure 1A. Briefly, the treatment administration schedule was the following: 4 cycles of i.p. FOLFIRINOX injections (Q3Dx4) prior the chemo-switch, followed by i.p. FOLFIRINOX injections every third day for the continuous FOLFIRINOX (FX) arm or i.p. PAD injections every second day for the PAD maintenance arm (FX→PAD). Overall survival was calculated as the time elapsed between AKC cells transplantation and mouse euthanasia reaching a pre-defined ethical endpoint. Body weight progression data are represented as the mean ± SD and referred to −20% weight loss (red dashed line Figure 2A) of the mean body weight of all mice at baseline. Mice were euthanized when an ethical endpoint was reached. Tumors and organs were then resected and fixed in cold 4% formaldehyde for 24 h and embedded in paraffin for histological analysis.

5-fluorouracil (NSC 19893), folinic acid (leucovorin), CC-115, irinotecan (CPT-11), oxaliplatin (L-OHP), olaparib (AZD2281, KU-00594), and VE-822 (VX970) were purchased from Selleckchem (Houston, TX, USA).

### 2.3. Magnetic Resonance Imaging

Magnetic resonance imaging was performed on a dedicated ultrahigh field 11.7T small animal system (BioSpec 117/16, Bruker Biospin, Billerica, MA, USA) equipped with a 9 cm gradient insert (BGA-S9) operating with ParaVision 6.01 software (Bruker Biospin) to assess tumor growth and metastasis burden. Animals were scanned prior to and after 10, 17, 19, and 21 days of treatment. All data were obtained with a four-channel receive-only surface coil placed anterior to the TA. Anesthesia was maintained using 1.5% isoflurane (Abbvie, North Chicago, IL, USA) and was adjusted to maintain a safe respiration rate of about 60 cycles per minute. The following MR scans were performed: 2D RARE in coronal and axial slice orientation with acquisition parameters as TE/TR = 23 ms/1500 ms, *r* = 90 × 90 × 500 µm³, and RARE factor = 8.

### 2.4. Cell Culture

*Atm^fl/fl^*; *LSL-Kras^G12D/+^*; *Ptf1a^Cre/+^* (AKC) cells were isolated from *Atm^fl/fl^*; *LSL-Kras^G12D/+^*; *Ptf1a^Cre/+^* mice and immortalized as described previously [16]. Cells were cultured in DMEM, containing 10% FBS (PAN Biotech, Aibendach, Germany) and P/S (100 IU/mL penicillin and 100 µg/mL streptomycin sulfate; ThermoFischer Scientific). All cells were propagated at 37 °C under 5% (v/v) CO_2_ atmosphere. All experiments were performed between passage 5 and 15. Mycoplasma tests were regularly performed using the Mycoprobe mycoplasma detection kit (R&D Systems, Minneapolis, MN, USA).

### 2.5. Migration Assay

For the transwell migration assay, 2 × 10^5^ cells were seeded in serum-free medium in the upper chamber of the transwells with an 8 µm pore size membrane (24-well format, Falcon, Corning) and complete medium in the lower chamber. After 24 h, cells were fixed with cold 4% formaldehyde and were stained with 5% Giemsa. Cells migrating to the membrane lower side were counted using ImageJ software (National Institutes of Health, Bethesda, MD, USA). Data are represented as the mean ± SD.

### 2.6. Immunohistochemistry

All histological experiments were performed as previously described [16]. The primary antibodies used were rabbit monoclonal antibodies against KI67 (1:250, ThermoFisher Scientific), H2AX p-S139 (1:400, clone 20E3, Thermo Fisher Scientific), and vimentin (1:500, clone D21H3, Cell Signaling Technology, Danvers, MA, USA); and rat monoclonal antibody against CK19 (1:100, Troma-III-s, Developmental Studies Hybridoma Bank, Iowa city, IA). Brightfield images were obtained using CFI Plan Apo 4×/0.2, CFI Plan Apo 10×/0.45, and CFI Plan Apo 20×/0.75 (Nikon, Minato City, Tokyo) objectives mounted on a BZ-9000 (Keyence, Osaka, Japan) microscope. Acquired pictures were subsequently analyzed using ImageJ software. Careful histological observations were made on 4 to 10 slides for each sample. KI67, H2AX p-S139, vimentin, and CK19-positive areas were quantified using ImageJ software (IHC image analysis toolbox, normalization of positive areas to the total surface to calculate the respective percentage occupied by positive staining per field). All quantifications were performed on at least three random pictures of all available orthotopic tumors (primary tumor: *n* ≥ 6 per condition) included in the study, and statistical analysis was performed among the different groups after comparison of each data set over the corresponding vehicle group. Data are represented as the mean ± SD.

### 2.7. Immunofluorescence

Cells were grown on glass coverslips, fixed in 4% formaldehyde at 4 °C, and permeabilized with 0.05% Tween20 for 15 min before immunofluorescence experiments. F-actin was stained with phalloidin-Atto565 (1:500; Sigma-Aldrich, Merck KGaA, Darmstadt, Germany). DAPI was contained within the ProLong Diamond Antifade mountant (ThermoFisher Scientific). Images were obtained at ambient temperature using CFI Plan Apo 4×/0.2, CFI Plan Apo 10×/0.45, and CFI Plan Apo 20×/0.75 (Nikon) objectives mounted on a BZ-9000 (Keyence) microscope. All analyzed pictures were carefully checked by eye to exclude artifacts and false positive areas. Data are represented as the mean ± SD.

### 2.8. Statistical Analysis

GraphPad Prism software (San Diego, CA, USA) was used for statistical analysis. For orthotopic assay survival, statistical significances were tested using log-rank (Mantel–Cox) tests. For the migration assays and immunostaining quantifications, statistical significances were tested using Student’s *t*-test (unpaired, two-tailed). Statistical significance in a contingency table was tested using Fisher’s exact test. Kaplan–Meier analysis was used for calculation of survival times. All tests were considered to be statistically significant when *p* < 0.05.

## 3. Results

### 3.1. Study Design

Based on previous findings and conducted dosage titrations [13], we established a synergistically operating therapy approach with the maximal possible HR and NHEJ inhibition in ATM-deficient murine PDAC cells. In this context, synergism between PARP, ATR, and DNA-PKcs inhibitors allowed the substantial lowering of single drug dosages while maintaining a highly potent cytotoxic effect [13]. We referred to this approach comprising the inhibition of **P**ARP (olaparib), **A**TR (VE-822), and **D**NA-PKcs (CC-115) as multi-DDR interference (mDDRi) and applied the latter regimen in a maintenance therapy setting following induction with the standard of care regimen FOLFIRINOX in a preclinical trial setting in analogy to the POLO trial. We referred to this mDDRi regimen as **PAD**. Tumors were induced upon orthotopic transplantation of ATM-deficient primary PDAC cells (***A****tm^fl/fl^; LSL-**K**ras^G12D/+^; **P**tf1a^Cre/+^*, **AKC**) [15,16]. Tumor growth was monitored repetitively over time using magnetic resonance imaging (MRI). The primary outcome of this preclinical trial was median overall survival (mOS) in the three arms comparing (i) vehicle (Veh) vs. (ii) maintained FOLFIRINOX (FX) vs. (iii) induction with four cycles (application every 3 days) of FOLFIRINOX followed by PAD maintenance therapy (FX→PAD) (Figure 1A). Secondary outcomes were metastatic load, body weight loss, and treatment associated damage to other organs.

### 3.2. Efficacy

The mOS was significantly longer with 28.5 days after FOLFIRINOX induction followed by PAD maintenance (FX→PAD) compared to the 24.5 days with maintained FOLFIRINOX (FX) therapy (*p* = 0.0193, HR 0.39, CI 95% 0.13–1.15). Similarly, both treatment arms significantly outperformed the placebo group (mOS 28.5 and 24.5 vs. 18.0 days; FX→PAD vs. vehicle: *p* = 0.0002; FX vs. vehicle: *p* = 0.0106) (Figure 1B). Remarkably, PAD maintenance allowed long-term survival up to 43 days in mice (Figure 1B). The increased mOS is nicely reflected in the MRI dynamics of individual mice over time, clearly visualizing improved disease control in the PAD maintenance (FX→PAD) arm therapy (Figure 1C,D). Tumor growth was assessed in analogy to RECIST 1.1 criteria in patients [26] and showed massive progression of the primary tumor on Day 17 compared to Day 10 (+41.1% increase) in the vehicle group, resulting in the rapid deterioration of every animal enrolled in this arm (Figure 1C). The maintained FOLFIRINOX treatment reduced tumor growth dynamics compared to the vehicle group (+20.7% vs. +41.1% increase of the primary tumor); however, it has been correlated with a high metastatic burden resulting in a profound alteration of the liver integrity as observed by MRI on Day 21 (Figure 1D, left column). Switch to PAD maintenance therapy did not fully prevent tumor growth, although it resulted in disease stabilization with 18.1% growth of the primary tumor on Day 21 and a remarkable complete remission of liver metastases compared to Day 10 (Figure 1D, right column).

### 3.3. Safety

The median time of treatment was 20 days (range 10–25 days) in the FOLFIRINOX (FX) group and 24 days (range 17–34 days) in the FOLFIRINOX induction followed by the PAD maintenance (FX→PAD) group. Mice were weighed daily throughout the trial, as shown in Figure 2A for the respective trial groups. As an ethical endpoint, a maximum weight loss of 20% compared to baseline was prespecified as the limit, which occurred in 37.5% of the mice who received FOLFIRINOX (FX) and similarly in 37.5% of the mice who received subsequent PAD maintenance (FX→PAD). Histological assessment of the liver and intestine (as usually most affected organs upon chemotherapy) did not reveal gross abnormalities. More specifically, no differences in proliferation patterns and no signs of increased DNA damage levels, illustrated by similar H2AX p-S139 signals, were observed among the different treatments (Figure 2B,C).

### 3.4. Phenotyping of Primary Tumors

**Figure 3:**
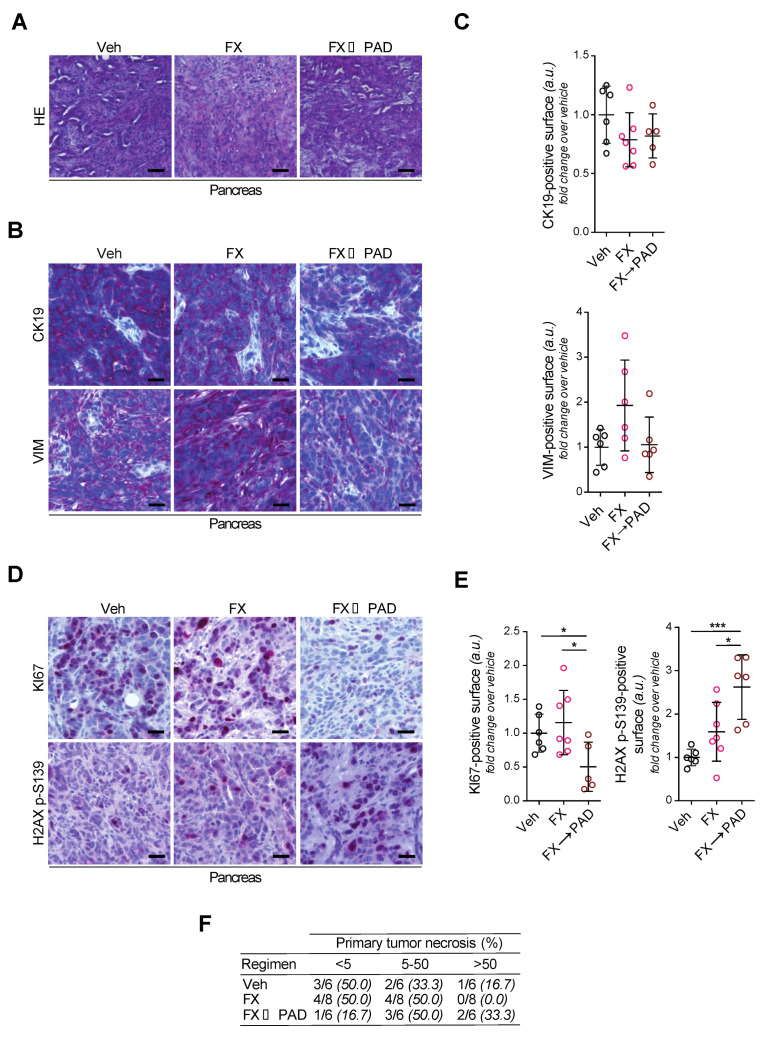
PAD maintenance therapy provokes DSB accumulation and compromises cell proliferation in ATM-deficient PDAC. (**A**) Histologic sections of resected pancreatic tumors from the orthotopic assay shown in Figure 1B, stained by hematoxylin–eosin. Scale bars represent 100 µm. (**B**) Immunohistochemistry staining for CK19 and vimentin, and (**C**) quantifications of CK19-positive and vimentin-positive surfaces in resected pancreatic tumors from the orthotopic assay shown in Figure 1B. Scale bars represent 50 µm. (**D**) Immunohistochemistry staining for KI67 and H2AX p-S139, and (**E**) quantifications of KI67-positive and H2AX p-S139-positive surfaces in resected pancreatic tumors from the orthotopic assay shown in Figure 1B. Scale bars represent 50 µm. (**F**) Contingency table comparing treatment regimen and necrotic surface. FX, FOLFIRINOX; PAD, PARP inhibitor/ATR inhibitor/DNA-PK inhibitor; Veh, vehicle. * *p* < 0.05, *** *p* < 0.001.

PDAC patients commonly either die from complications of the primary tumor or during disease progression from metastatic dissemination [27]. Histopathological characterization of primary cancers found aggressive, less differentiated PDACs displaying a high expression of vimentin, in line with the previously described epithelial–mesenchymal transition induced by ATM deletion [15] (Figure 3A–C). Interestingly, FOLFIRINOX-treated primary tumors appeared even more de-differentiated, as illustrated by the presence of more vimentin-positive tumor cells (Figure 3B,C). Tumors of the vehicle-treated group were most prominent in size as outlined by MRI follow-up (Figure 1C,D). They revealed similarly high proliferative capacities as the FOLFIRINOX-treated tumors albeit the endpoint was reached earlier in vehicle-treated animals, and the FOLFIRINOX dosage regimen has been previously validated [25] (Figure 3D,E). This significantly contrasts the PAD maintenance-treated (FX→PAD) tumors that virtually lost proliferative capacity (Figure 3D,E). Conversely, the FOLFIRINOX induction followed by the PAD maintenance (FX→PAD) group showed the highest level of H2AX p-S139-positive signal (as the correlation for DNA damage), followed by the tumors in the FOLFIRINOX (FX) and the vehicle arm (Figure 3E). In line, the highest ratio of necrosis was observed in the PAD maintenance arm (FX→PAD), attesting to a higher cytotoxic effect in this group (Figure 3F). Altogether, the greater amount of DSBs indicates high levels of genotoxic stress as the primary driver of cytotoxicity in the PAD maintenance (FX→PAD)-treated mice.

### 3.5. Tumor Invasion and Dissemination in the Respective Study Arms

**Figure 4:**
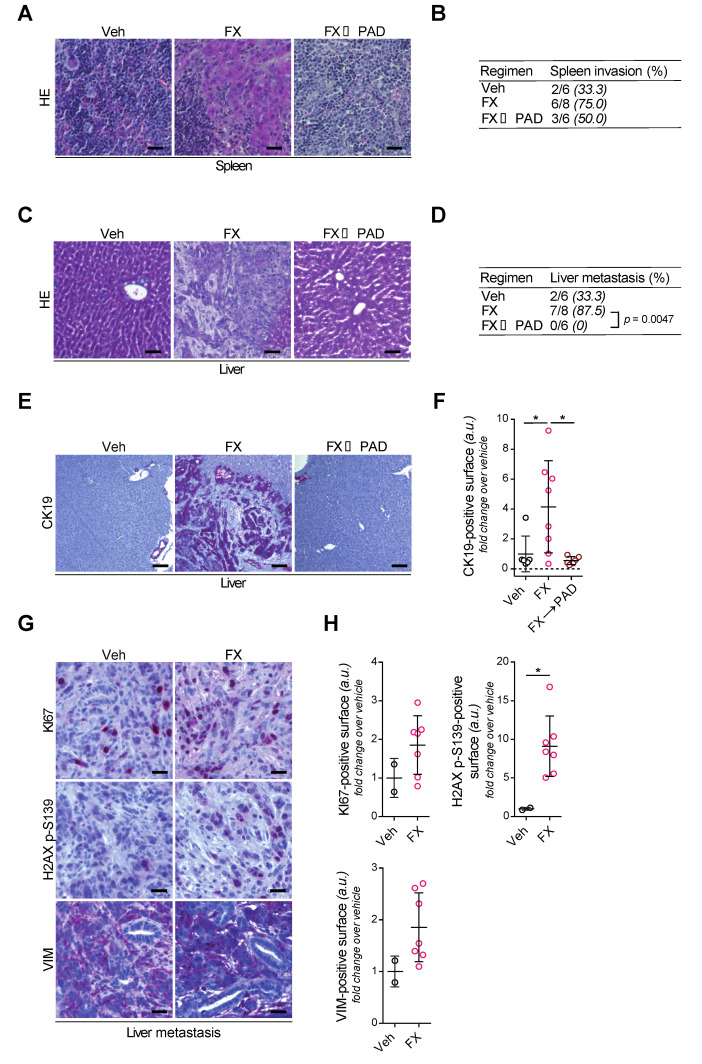
PAD maintenance therapy reduces metastatic burden in ATM-deficient PDAC. (**A**) Histologic sections of resected spleen from the orthotopic assay shown in Figure 1B, stained by hematoxylin–eosin. Scale bars represent 50 µm. (**B**) Contingency table comparing treatment regimen and spleen invasion. (**C**) Histologic sections of resected livers from the orthotopic assay shown in Figure 1B, stained by hematoxylin–eosin. Scale bars represent 100 µm. (**D**) Contingency table comparing treatment regimen and presence of liver micrometastasis. (**E**) Immunohistochemistry staining for CK19 and (**F**) quantifications of CK19-positive surface in resected livers from the orthotopic assay shown in Figure 1B. Scale bars represent 250 µm. (**G**) Immunohistochemistry staining for KI67 and H2AX p-S139, and (**H**) quantifications of KI67-positive and H2AX p-S139-positive surfaces in resected livers from the orthotopic assay shown in Figure 1B. Scale bars represent 50 µm. FX, FOLFIRINOX; PAD, PARP inhibitor/ATR inhibitor/DNA-PK inhibitor; Veh, vehicle. * *p* < 0.05.

Liver metastases were particularly evident in MRI images taken from mice in the FOLFIRINOX arm, while none were found in the other arms at Day 21 of the respective treatment arms (Figure 1D). Remarkably, evident metastases in the liver after FOLFIRINOX induction therapy at Day 10, fully disappeared when therapy was switched to PAD until day 21 (Figure 1D). In fact, aggressive local infiltration of vital structures (e.g., stomach, small intestine, spleen, and liver) was most evident in vehicle-treated PDACs, indicating primary tumors as the leading cause of death in this group (Figure 1C). This prompted us to assess metastasis development more systematically. Spleen invasion by AKC tumor cells occurred less often in 50% (3/6 mice) of FX→PAD-treated mice when compared to 75% of mice (6/8 mice) treated with FOLFIRINOX, illustrating their decreased ability to invade adjacent organs (Figure 4A,B). Furthermore, histological screening upon hematoxylin–eosin and CK19 immunostaining of liver sections revealed a complete absence of micrometastasis in the PAD maintenance (FX→PAD) group as compared to the FOLFIRINOX-only (FX) group (Figure 4C–F), in line with the MRI findings. Similar to the primary tumors, metastasis arising under FOLFIRINOX exhibited a trend in favor of higher proliferation levels, expressed vimentin, and accumulated DSB as depicted by increased H2AX p-S139-positive signaling (Figure 4G,H). Additionally, the careful histological assessment of liver sections confirmed the low metastatic content of the vehicle-treated mice (2/6 mice; Figure 4C,D), as highlighted by the MRI monitoring (Figure 1C). In line with previous reports from highly aggressive PDAC models [28], vehicle-treated mice died as a result of ultra-fast uncontrolled tumor growth lacking the ability of pronounced metastatic tumor dissemination. Thus, pancreatic tumor cells lacking *Atm* expression might undergo an evolutionary process upon long-term exposure to components of the FOLFIRINOX regimen, most likely by selecting for more aggressive subclones.

### 3.6. FOLFIRINOX Selects Aggressive Subclones Erased by mDDRi

**Figure 5:**
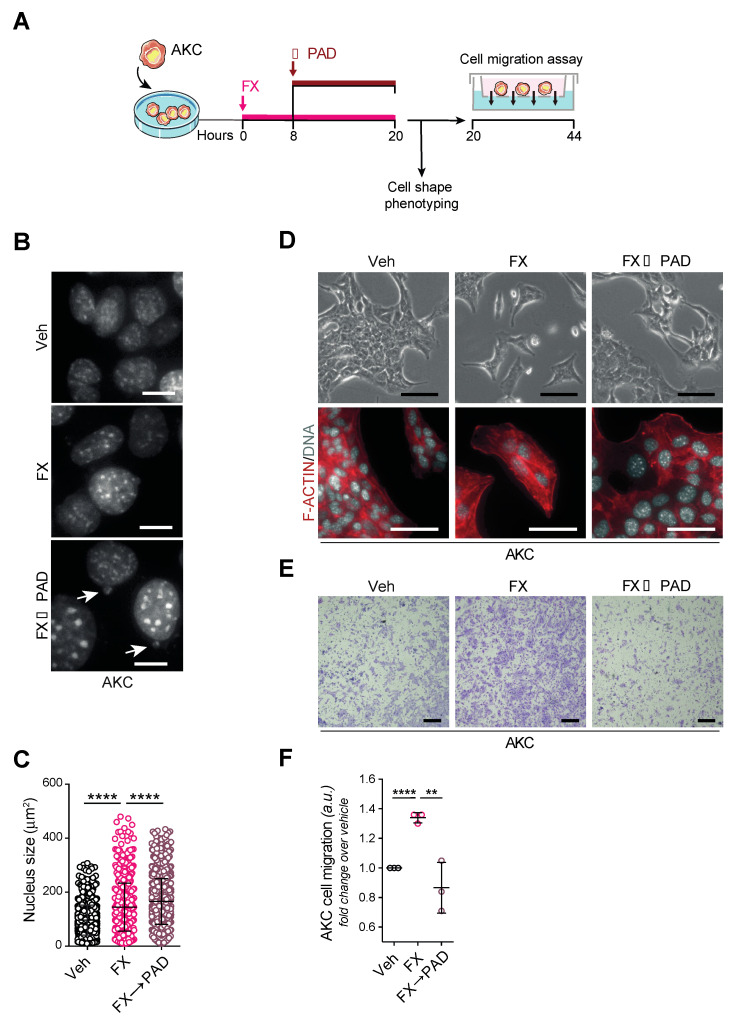
FOLFIRINOX therapy selects for more aggressive subclones in vitro. (**A**) Schematic representation of the cell phenotyping shown in (**B**–**D**) and the cell migration assay shown in (**E**). (**B**) Direct fluorescence staining of DNA by DAPI (white) in *Atm^fl/fl^; LSL-Kras^G12D/+^; Ptf1a^Cre/+^* (AKC) cells treated or not with FOLFIRINOX (folinic acid, 5-fluorouracil, irinotecan, and oxaliplatin) and sequentially with FOLFIRINOX and with olaparib (PARPi, 1 µM), VE-822 (ATRi, 20 nM) and CC-115 (DNA-PKi, 30 nM) in combination (PAD, PARPi/ATRi/DNA-PKi), for 20 h as shown in (**A**). White arrows show micronuclei (M). Scale bars represent 10 µm. (**C**) Nucleus size quantification in AKC cells treated or not with FOLFIRINOX and sequentially with FOLFIRINOX and with PAD as in (**B**). (**D**) Representative images (upper panels) and direct fluorescence staining of DNA by DAPI (white) and of cortical actin by phalloidin-Atto565 (red) (lower panels) in AKC cells treated or not with FOLFIRINOX and sequentially with FOLFIRINOX and with PAD as in (**B**). Scale bars represent 100 µm. (**E**) Transwell migration assay performed with AKC cells treated or not with FOLFIRINOX and sequentially with FOLFIRINOX and with PAD as in (**B**), and (**F**) quantifications of cell migration. Scale bars represent 250 µm. Veh, vehicle. ** *p* < 0.01; **** *p* < 0.0001.

To assess this observation in more detail, we exposed AKC cell lines toward sublethal dosage of either FOLFIRINOX or FOLFIRINOX followed by sequential PAD treatment and investigated cell shape as well as migration features (Figure 5A). DNA staining revealed nucleus abnormalities, including a significant increase of the nucleus size associated with the emergence of micronuclei upon sequential treatment compared to FOLFIRINOX (FX) treatment alone (Figure 5B,C). Interestingly, the sole FOLFIRINOX exposure further raised the mesenchymal phenotype of the AKC cells to an even more elongated shape and scattered distribution (Figure 5D). Conversely, the PAD maintenance regimen appeared to be correlated with a baseline mesenchymal phenotype less distinctly (Figure 5D). To probe this phenotypic observation with functional assays, we studied various treatment regimens in migration assays. Again, the sole FOLFIRINOX exposure led to an increase in the AKC cells’ migratory properties, an observation diminished in the sequential treatment algorithm involving the mDDRi regimen PAD (Figure 5E,F). Of note, no differences in cell viability were observed among FX and FX→PAD treatments (data not shown), corroborating that the decreased migration upon FX→PAD treatment did not result from increased cytotoxicity. Thus, albeit highly efficient in *Atm*-null pancreatic cancer cells, the sole FOLFIRINOX treatment selects for more aggressive subclones, which can be partly erased by the following PAD maintenance treatment, an in vitro observation supporting our in vivo data.

## 4. Discussion

The POLO trial provided a milestone in targeted therapy of PDAC albeit tailored to a minimal subgroup of around 3–6% of germline *BRCA1/2*-mutated patients with pancreatic cancer [7,29]. Previous work from our group suggests that other more often mutated DDR genes, involved in homologous recombination (HR) mediated DSB repair as *Atm*, can similarly ascribe sensitivity toward PARP inhibitor treatment [15,16]. Based on systematic testing, we hypothesized that other compensatory operating pathways could sustain cellular viability upon PARP inhibition in *Atm*-null PDAC, establishing PAD as a valuable alternative [16]. Albeit accumulating evidence suggests FOLFIRINOX, or better said, a platinum-based regimen, to be superior to others in case of a DDR-defective PDAC [11], the optimal therapy sequence and strategy remains unclear for now [30,31]. In this preclinical trial, we extend the format of the POLO study in various ways: (i) we validated the POLO design in *Atm*-defective PDAC, (ii) we dissected the consequences of distinctive application regimen, and (iii) describe a “hit hard and early but preserve smart” strategy compared to the standard of care chemotherapy strategy. Likewise, we confirmed the overall effectivity of FOLFIRINOX as proven previously in a preclinical trial setting [25], but disturbingly found evidence of FOLFIRINOX-driven Darwinian evolution selecting for highly aggressive escaper clones at least in this preclinical setting. Vice versa, sensitizing the tumor with the latter regimen followed by mDDRi interrogation appeared to reduce this selection as indicated by reduced metastatic seeding and subsequently improved survival.

Generally, primary and secondary evolving chemoresistance limits treatment efficacy in PDAC, and our results nicely illustrate the consequences on HR-deficient PDAC. Usually, the selection process not only allows survival of regular PDAC cells but instead selects for better adopted, more aggressive subclones [32]. The more efficient the primary regimen operates, the more aggressive arising subclones could be selected that may also switch in their subtype identity [33], as suggested by our observation following sole FOLFIRINOX treatment. In contrast, targeted therapies are usually less efficient but operate smarter and more specific. However, they still have proven little benefit in PDAC over the last decade [3], unless olaparib showed the first significant clinical response in the Phase III POLO trial [10]. In a recent study, we have shown that PAD tri-therapy acts synergistically and synthetically lethal on ATM-deficient cells both in mouse and human model systems [13]. In line, it was previously shown that ATM-deficient tumors rely on DNA-PKcs function [20]. As well, the inhibition of ATR was described to promote lethality in proliferating cells [34]. ATR and DNA-PKcs inhibitions were both reported to be highly potent therapies as sole treatment [35], but unfortunately associated with serious side effects. Nevertheless, the exploitation of inhibition synergism allowed the elaboration of a highly efficient but tolerable synergistic cocktail against ATM-deficient PDAC [13]. Mechanistically, we reported that PAD combination exploits the effect of unscheduled origin firing due to ATR inhibition and the persistence of DSB due to inhibition of ATM and DNA-PKcs. In this context, the excessive exacerbation of replication stress and DNA damage finally lead to genomic instability and cell death after the induction of fatal pathways, such as apoptosis [13]. Therefore, the approach of our study to potentially extend the POLO design to other DDR mutated PDAC widens the preclinical basis to a generalized treatment of HR-defective PDAC. Remarkably, the maintenance therapy with PAD allowed several prolonged surviving mice compared to maintained FOLFIRINOX therapy. Moreover, it ablated already present metastases in the liver after FOLFIRINOX induction in line with the abrogation of escaper clones being more invasive. Therefore one might speculate that the triple inhibition of PARP, ATR, and DNA-PKcs in an HR deficient context could prevent the observed selection process upon prolonged FOLFIRINOX, probably by increasing DNA damage beyond a tolerable threshold due to the sensitizing effects of FOLFIRINOX [19]. This ablation of any escaper clones is likely responsible for reduced metastatic burden [36]. Moreover, one can speculate that the therapy with oxaliplatin and the DNA topoisomerase inhibitor irinotecan that have both proven to be effective in genetically unstable PDAC could select for tumor cell clones with a higher tolerance of genomic instability due to their innate mode of action. Accordingly, our strategy with maintenance mDDRi seems to be effective by pushing genomic instability beyond a tolerable threshold, to erase escaper clones. This allows speculation that FOLFIRINOX specifically leaves clones behind, which are particularly permissive for such a targeted PAD attack. Collectively, we extend the POLO design to *Atm*-null PDAC and favor a “hit hard and early but preserve smart” strategy using mDDRi.
